# Radiological and pathological analysis of the galaxy sign in patients with pulmonary mucosa‐associated lymphoid tissue (MALT) lymphoma

**DOI:** 10.1111/1759-7714.15029

**Published:** 2023-07-06

**Authors:** Yeongran Song, Yeoun Eun Sung, Kyongmin S. Beck, Suyon Chang, Jung Im Jung, Gyeong Sin Park

**Affiliations:** ^1^ Department of Radiology, Seoul St. Mary's Hospital, College of Medicine The Catholic University of Korea Seoul Republic of Korea; ^2^ Department of Hospital Pathology, Seoul St. Mary's Hospital, College of Medicine The Catholic University of Korea Seoul Republic of Korea

**Keywords:** computed tomography, galaxy sign, MALT lymphoma, marginal zone B cell lymphoma

## Abstract

**Background:**

Pulmonary mucosa‐associated lymphoid tissue (MALT) lymphoma sometimes presents as large pulmonary nodules composed of small nodular opacities (galaxy sign) on computed tomography (CT). The aim of this study was to assess the presence, usefulness, and pathological characteristics of the galaxy sign on CT of pulmonary MALT lymphoma.

**Methods:**

From January 2011 to December 2021, chest CTs of 43 patients with pulmonary MALT lymphoma were reviewed by two radiologists for the galaxy sign and various other findings. Interreader agreement to characterize the galaxy sign and factors associated in making a correct first impression on CT prior to pathological diagnosis were assessed. Resected specimens were reviewed by two pathologists, and the proportion of peripheral lymphoma infiltrates was compared between lesions with and without the galaxy sign.

**Results:**

Of 43 patients, 22 patients (44.2%) showed the galaxy sign (*κ* = 0.768, *p* < 0.0001). The galaxy sign (*p* = 0.010) was associated with making a correct first impression on CT prior to pathological diagnosis. On pathological examination, lesions showing the galaxy sign on CT demonstrated a significantly higher proportion of peripheral lymphoma infiltrates (*p* = 0.001).

**Conclusion:**

The galaxy sign can be seen on CT of pulmonary MALT lymphoma with a higher proportion of peripheral lymphoma infiltrates and may be useful in making a correct diagnosis of pulmonary MALT lymphoma.

## Graphical abstract

Pulmonary mucosa‐associated lymphoid tissue (MALT) lymphoma sometimes presents as large pulmonary nodules composed of small nodular opacities (galaxy sign) on CT. The galaxy sign can be seen on CT of pulmonary MALT lymphoma with a higher proportion of peripheral lymphoma infiltrates and may be useful in making a correct diagnosis of pulmonary MALT lymphoma. Knowing that pulmonary MALT lymphoma can also present as the galaxy sign on CT would be useful in making a differential diagnosis on CT.

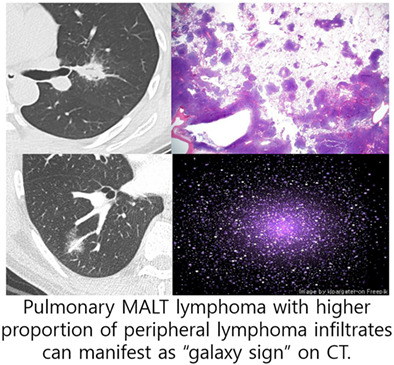

## INTRODUCTION

Mucosa‐associated lymphoid tissue (MALT) lymphoma is a low‐grade, extranodal marginal zone B cell lymphoma with a favorable prognosis, representing up to 8% of all lymphomas.^1^ MALT lymphoma can involve any mucosal sites in the body, usually the gastrointestinal tract but also the lungs. Pulmonary MALT lymphoma accounts for 10%–15% of all MALT lymphomas, but is the most common subset of primary pulmonary lymphomas, accounting for approximately 60%.^2^ There are several previous studies on the radiological appearances of pulmonary MALT lymphoma, which exhibit diverse and broad categories of imaging findings.^3^, ^4^, ^5^, ^6^, ^7^, ^8^, ^9^, ^10^, ^11^ Reported imaging findings include single or multiple masses, nodules, or consolidations with air bronchogram, enhancing vessels within the masses or consolidation (computed tomography [CT] angiogram sign), or halo sign.^3^, ^4^, ^5^, ^6^, ^7^, ^8^, ^9^, ^10^, ^11^ However, these reported imaging findings are not specific enough and overlap with other lung diseases, such as adenocarcinoma, metastasis, or pneumonia. In reality, MALT lymphoma is often difficult to diagnose before pathological verification. Accurate and timelier differentiation of MALT lymphoma from other diseases would be helpful to the patients, considering the different clinical behaviors and treatments. In our daily practice, we have experienced MALT lymphoma presenting as large pulmonary nodules composed or surrounded by small nodular opacities on CT, similar to the “galaxy sign” seen in sarcoidosis or tuberculosis.^12^, ^13^, ^14^ The galaxy sign represents an irregularly margined nodule formed by confluence of multiple smaller nodules, more concentrated in the center than at the periphery.^14^ Knowing that the galaxy sign can be seen in MALT lymphoma would be helpful in the differential diagnosis of patients who would otherwise be evaluated only for sarcoidosis or tuberculosis. Also, it could be useful in differentiating MALT lymphoma from other diseases prior to pathological verification. The purpose of this study was to determine the presence, usefulness, and pathological characteristics of the galaxy sign in patients with pulmonary MALT lymphoma.

## METHODS

### Study population

After reviewing the image archiving communication system and pathological records at our hospital from January 2011 to December 2021, 43 patients with pathologically confirmed pulmonary MALT lymphoma and available chest CT images were included in this study. The following clinical data were obtained from the electronic medical records: age, sex, presence of extrapulmonary involvement at the time of pathological verification, and method of pathological verification.

### Image analysis

All chest CT scans were independently and retrospectively reviewed by two thoracic radiologists (Kyongmin S. Beck and Suyon Chang, each with 7 and 6 years, respectively, of clinical experience in thoracic radiology) to assess the presence of the galaxy sign and various other radiological features of pulmonary MALT lymphoma. When there was a disagreement between two radiologists, a conclusion was reached by consensus.

Based on CT findings, pulmonary MALT lymphomas were first categorized into three major patterns: (1) mass/nodule, (2) consolidation and (3) both mass/nodule and consolidation. Mass/nodule was defined as a rounded well‐defined or moderately well‐defined opacity, with a nodule being up to 3 cm in diameter and a mass being greater than 3 cm in diameter.^15^ Areas of consolidation were defined as a homogeneous increase in pulmonary parenchymal attenuation that obscures the margins of vessels and airway walls.^15^ The number of lesions on CT was also assessed.

**FIGURE 1:**
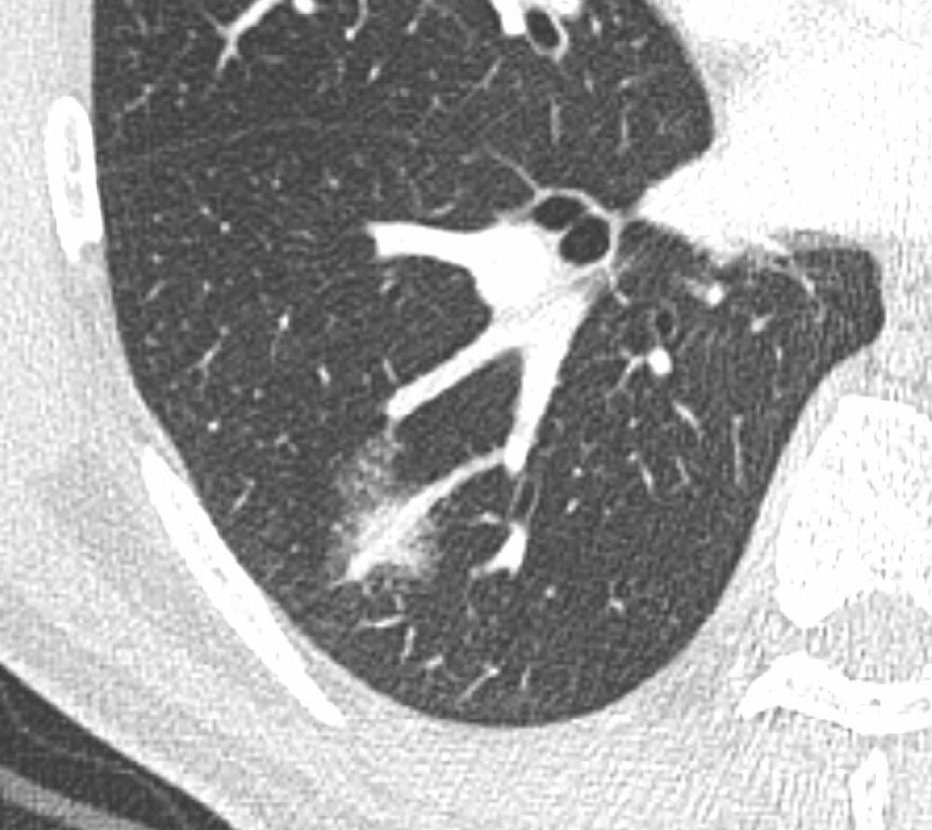
Axial chest computed tomography (CT) of a 58‐year‐old male with pulmonary mucosa‐associated lymphoid tissue (MALT) lymphoma shows a confluent nodule with multiple tiny peripheral nodules emanating from the margins of the central nodule in the right lower lobe, known as the “galaxy sign”.

The presence of the galaxy sign (Figure 1), which was defined as the appearance of confluent nodule with multiple small peripheral nodules emanating from the margins of the central nodule,^12^ was then assessed. In addition, following CT features, which were selected based on previous studies,^3^, ^4^, ^5^, ^6^, ^7^, ^8^, ^9^, ^10^, ^11^ were also analyzed: air bronchogram, bronchocentric distribution, ground‐glass opacity (GGO) component, perilesional spicules, separate interlobular septal thickening, and cavitation. Well‐known terms such as air bronchogram, bronchocentric distribution, GGO, interlobular septal thickening, and intralesional cavity, were defined using the Fleischner Society glossary.^15^ Perilesional spicules were defined as nodular or smooth interstitial thickening around a pulmonary lesion, seen as irregular spiculations. Separate interlobular septal thickening was defined as interlobular septal thickening separate from a pulmonary lesion. Intrathoracic mediastinal lymph nodes greater than 1 cm in short‐axis diameter on CT were classified as enlarged. Definitions of terms with example images were made into a separate document to be used for image analysis (supplementary material: Data S1). When lesions were multiple, presence of the features in any of the lesions was considered present for said features. We then evaluated the diagnostic accuracy using the first impression at chest CT prior to tissue confirmation based on the formal radiology report.

### Pathological analysis

Cases in which representative lung lesions were surgically resected were pathologically analyzed. Surgically resected lung specimens were routinely fixed in 10% formalin solution, followed by paraffin embedding, 4 μm sectioning, and hematoxylin & eosin (H&E) staining.

Immunohistochemical staining with CD3 (Dako, rabbit polyclonal antibody), CD20 (Dako, mouse monoclonal antibody; L26), Ki‐67 (Ventana Medical Systems Inc., rabbit monoclonal antibody; 30–9) and pancytokeratin (Dako, mouse monoclonal antibody; AE1/AE3; 1:400) for diagnosis were performed. All H&E and immunohistochemically stained slides were reviewed by two pathologists (Kyongmin S. Beck and Yeoun Eun Sung, each with 20 and 4 years, respectively, of clinical pathology).

Molecular testing for diagnosis was performed in only a subset of the included cases, as these tests are known to be useful in some problematic cases, rather than routinely recommended in all cases.^16^ Molecular tests performed included IgH gene rearrangement (4 cases), MALT1 gene fluorescence in situ hybridization (FISH) (1 case), and kappa/lambda in situ hybridization (1 case). Monoclonality was confirmed by IgH gene rearrangement, break‐apart was confirmed by FISH, and light chain restriction was observed by kappa/lambda in situ hybridization. Detailed methods of molecular testing are described in the supplementary material: Data S1.

**FIGURE 2:**
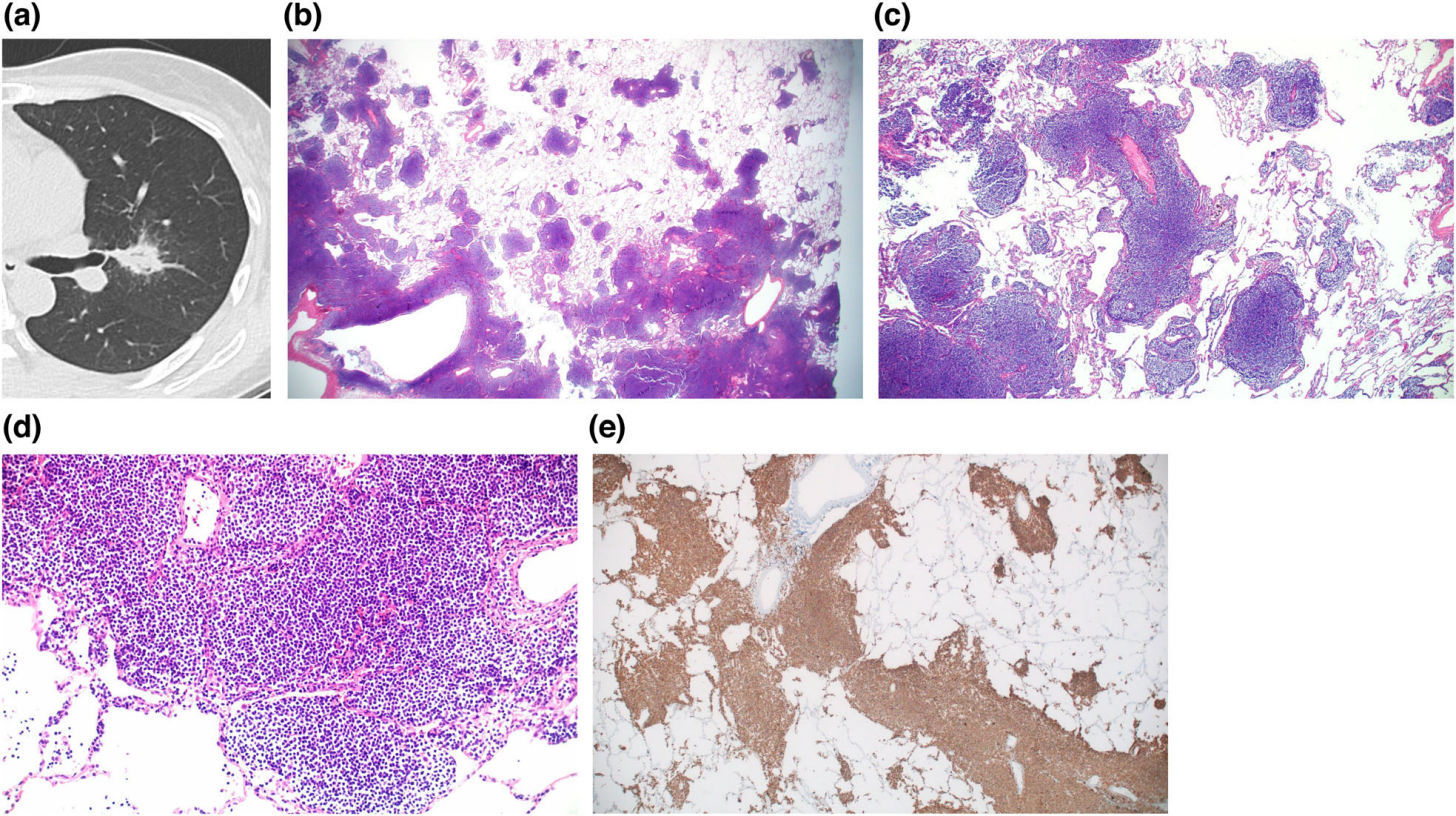
Pulmonary mucosa‐associated lymphoid tissue (MALT) lymphoma in a 50‐year‐old female. (a) Axial chest computed tomography (CT) image shows a confluent peribronchial nodule with multiple tiny peripheral nodules, indicating the galaxy sign, in the left upper lobe. (b) Microscopic examination of the resected surgical specimen at a low power field (x 12.5) shows a greater extent of the peripheral area compared to the central area. (c) A more magnified view (x 40) of the specimen shows peripherally located neoplastic infiltrates consisted of nodular lesions of varying sizes and shapes and occasional reactive germinal centers, with variable distance among the individual infiltrates. (d) At a higher power field, monotonous neoplastic lymphocytes comprising the peripheral nodule are observed. (e) CD20 immunohistochemical staining of the specimen highlights neoplastic B cells.

**FIGURE 3:**
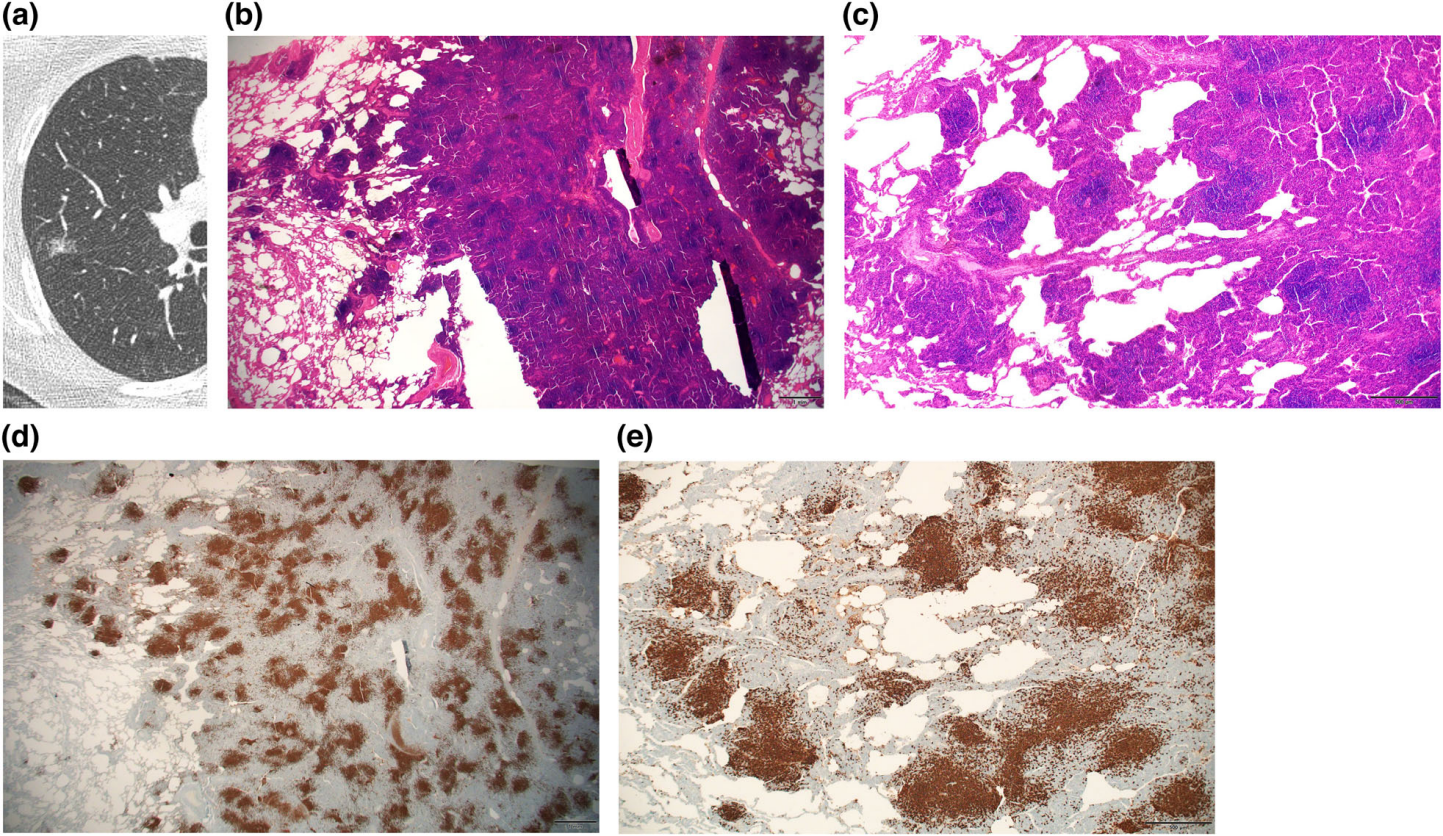
Pulmonary mucosa‐associated lymphoid tissue (MALT) lymphoma in a 63‐year‐old female. (a) Axial chest computed tomography (CT) image shows a confluent peribronchial nodule with multiple tiny peripheral nodules, indicating the galaxy sign, in the right upper lobe. (b) Microscopic examination of the resected surgical specimen at a low power field (× 12.5), shows a greater extent of peripheral area compared to the central area. (c) At a higher power field, peripherally located neoplastic infiltrates consisting of nodular lesions of varying sizes and shapes are observed. (d, e) Neoplastic B lymphocytes are highlighted by CD20 immunohistochemistry.

**FIGURE 4:**
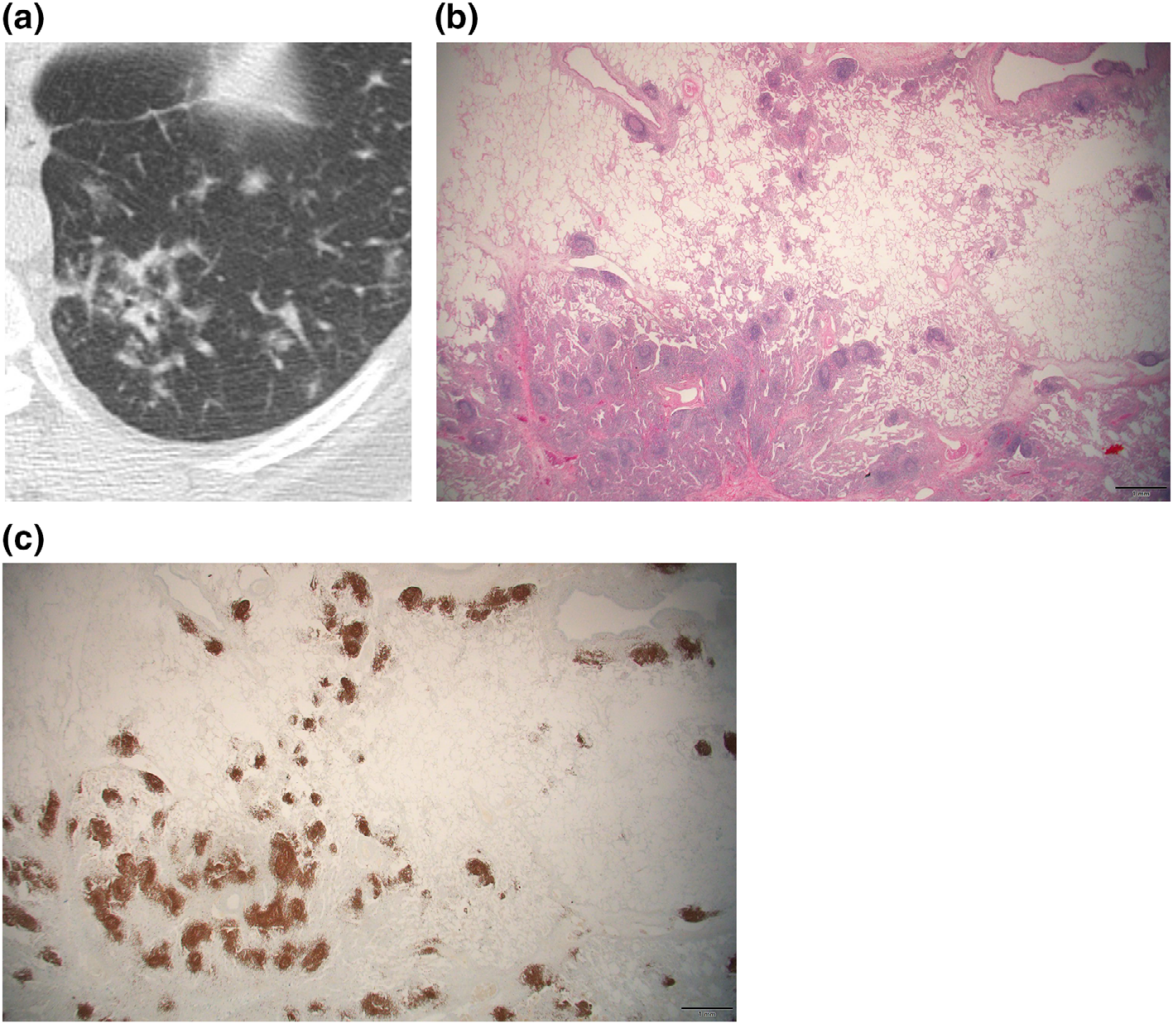
Pulmonary mucosa‐associated lymphoid tissue (MALT) lymphoma in a 40‐year‐old male. (a) Axial chest computed tomography (CT) image shows multiple tiny coalescent nodules along the bronchovascular bundles. (b) Microscopic examination of the resected surgical specimen at a low power field shows neoplastic lymphocytic infiltrates in the peribronchiolar area adjacent to the main tumor mass. (b) Neoplastic B lymphocytes are highlighted by CD20 immunohistochemistry.

The lymphomatous infiltrate consists of varying proportions of small lymphocytes, centrocyte‐like cells, monocytoid B cells and plasma cells, frequently surrounding reactive germinal centers. Each lymphoma lesion in the available surgical specimen was examined for the center showing solid area with alveolar destruction and periphery where neoplastic cells track along the bronchovascular bundles in a skip‐lesion pattern (Figures 2, 3, 4). The proportion of the center and the periphery in percentage, decided in consensus by two pathologists, were recorded. The border of the peripheral lesion was determined by the outermost lymphoma infiltrate around the bronchovascular bundle. Then the proportion of the periphery was compared between those with the galaxy sign and those without the galaxy sign. When there were multiple lesions on a surgical specimen, one radiologist (Kyongmin S. Beck) checked the CT for the presence of the galaxy sign for each lesion.

### Statistical analysis

Statistical analysis was performed using SPSS software version 24.0 (SPSS Inc.). Interreader agreement to recognize the galaxy sign was assessed using the results of independent review before reaching a consensus and by kappa statistical analysis. The κ values were interpreted as follows: less than 0.20, poor agreement; 0.21–0.40, fair agreement; 0.41–0.60, moderate agreement; 0.61–0.80, good agreement; and 0.81–1.00, very good agreement. Univariable logistic regression analyses were performed to determine factors associated with correct first impression at chest CT prior to tissue confirmation. A *p*‐value of less than 0.05 was considered statistically significant. The comparison of proportion of the periphery on pathological examination between those with the galaxy sign and those without the galaxy sign were analyzed using the student's *t*‐test.

## RESULTS

### Patient characteristics and CT findings

Of 43 patients (23 male, mean age 57.7 ± 13.2 years) included in the study, 22 patients (51.2%) showed the galaxy sign. The interreader agreement for the presence of the galaxy sign was 0.768 (*p* < 0.0001), which is interpreted as moderate agreement.

Pathological verification was done using lung biopsy, bronchoscopic biopsy, surgical resection, and both lung biopsy and surgical resection in 13, three, 24, and three patients, respectively. A total of eight patients (18.6%) had extrapulmonary involvement of MALT lymphoma at the time of pathological verification.

CT findings of pulmonary MALT lymphoma are shown in Table 1. A total of 29 patients (67.4%) demonstrated multiple lesions on CT. A total of 28 patients (65.1%) presented with a mass/nodule type on CT; 10 patients (23.3%) and five patients (11.6%), respectively, presented with a consolidation type and a combination of both mass/nodule and consolidation types on CT. The prevalence of CT features was as follows: Air bronchogram (*n* = 39, 90.7%), perilesional spicules (*n* = 34, 79.1%), bronchocentric distribution (*n* = 32, 74.4%), GGO component (*n* = 21, 48.8%), intralesional cavity (*n* = 10, 23.3%), enlarged lymph nodes (*n* = 6, 14.0%) and separate interlobular septal thickening (*n* = 5, 11.6%).

**TABLE 1:** Chest CT findings of pulmonary MALT lymphoma.

Chest CT findings	Total (*n* = 43)
Number of lesions	
Single	14 (32.6%)
Multiple	29 (67.4%)
Pattern	
Nodule/mass only	28 (65.1%)
Consolidation only	10 (23.3%)
Both nodule/mass and consolidation	5 (11.6%)
Associated features	
Air bronchogram	39 (90.7%)
Perilesional spicules	34 (79.1%)
Bronchocentric distribution	32 (74.4%)
Galaxy sign	22 (51.2%)
GGO component	21 (48.8%)
Intralesional cavity	10 (23.3%)
Lymphadenopathy (>1 cm)	6 (14.0%)
Separate interlobular septal thickening	5 (11.6%)
Correct first impression	17 (39.5%)

Abbreviations: CT, computed tomography; GGO, ground‐glass opacity, MALT, mucosa‐associated lymphoid tissue.

Correct first impression at chest CT prior to tissue confirmation occurred in 17 patients (39.5%). Primary lung cancer (*n* = 12, 27.9%) was the most common radiological misdiagnosis, followed by organizing pneumonia (*n* = 6, 14.0%), chronic infection, including tuberculosis (*n* = 6, 14.0%), metastasis (*n* = 1, 2.3%) and sarcoidosis (*n*= 1, 2.3%).

Univariable logistic regression analyses were performed to analyze the characteristics associated with making a correct first impression on CT (Table 2). The galaxy sign (*p* = 0.010, odds ratio [OR] = 6.14; 95% confidence interval [CI]: 1.54, 24.44), the presence of extrapulmonary involvement (*p* = 0.012, OR = 17.5; 95% CI: 1.90, 161.12), multiple lesions (*p* = 0.012, OR = 16.0; 95% CI: 1.84, 138.96), intralesional cavity (*p* = 0.033, OR = 5.37; 95% CI: 1.15, 25.11), bronchocentric distribution (*p* = 0.037, OR = 10.0; 95% CI: 1.14, 87.52), and consolidation type (*p* = 0.049, OR = 3.75; 95% CI: 1.00, 14.02) were factors associated with making a correct first impression on CT.

**TABLE 2:** Univariable logistic regression analysis for correct first impression on CT prior to pathological diagnosis.

Chest CT findings	Correct first impression (*n* = 17)	Incorrect first impression (*n* = 26)	OR (95% CI)	*p*‐value
Number of lesions				
Single	1 (5.9%)	13 (50.0%)	Ref	**0.012** *
Multiple	16 (94.1%)	13 (50.0%)	17.0 (1.84–138.96)	
Pattern				
Mass/nodule	12 (70.6%)	21 (80.8%)	0.57 (0.137–2.38)	0.442
Consolidation	9 (52.9%)	6 (23.1%)	3.75 (1.00–14.02)	**0.049** *
Other CT features				
Galaxy sign	13 (76.5%)	4 (15.4%)	6.14 (1.54–24.44)	**0.010** *
Air bronchogram	17 (100.0%)	22 (84.6%)	>999.999 (0.00‐)	0.999
Perilesional spicules	16 (94.1%)	18 (69.2%)	7.11 (0.80–63.24)	0.079
Bronchocentric distribution	16 (94.1%)	16 (61.5%)	10.0 (1.14–87.52)	**0.037** *
GGO component	8 (47.1%)	13 (50.0%)	0.89 (0.26–3.02)	0.850
Intralesional cavity	7 (41.2%)	3 (11.5%)	5.37 (1.15–25.11)	**0.033** *
Lymphadenopathy (>1 cm)	3 (17.6%)	3 (11.5%)	1.64 (0.29–9.29)	0.574
Separate interlobular septal thickening	2 (11.8%)	3 (11.5%)	1.02 (0.15–6.79)	0.982
Extrapulmonary involvement	7 (41.2%)	1 (3.8%)	17.5 (1.90–161.12)	**0.012** *

Abbreviations: CI, confidence interval; CT, computed tomography; GGO, ground‐glass opacity; OR, odds ratio; Ref, indicates the reference category for calculation of odds ratio; SD, standard deviation.

* *p* < 0.05 is indicated in bold.

### Pathological findings

Of 28 patients who had undergone surgical resection, pathological analysis was done with 25 available resected lung specimens. The other three patients had undergone surgical resection at other institutions and did not have resected lung specimens at our institution to be reviewed. Of 25 resected lung specimens, 33 lymphoma lesions were examined, and there were 15 lymphoma lesions that showed the galaxy sign on CT.

**FIGURE 5:**
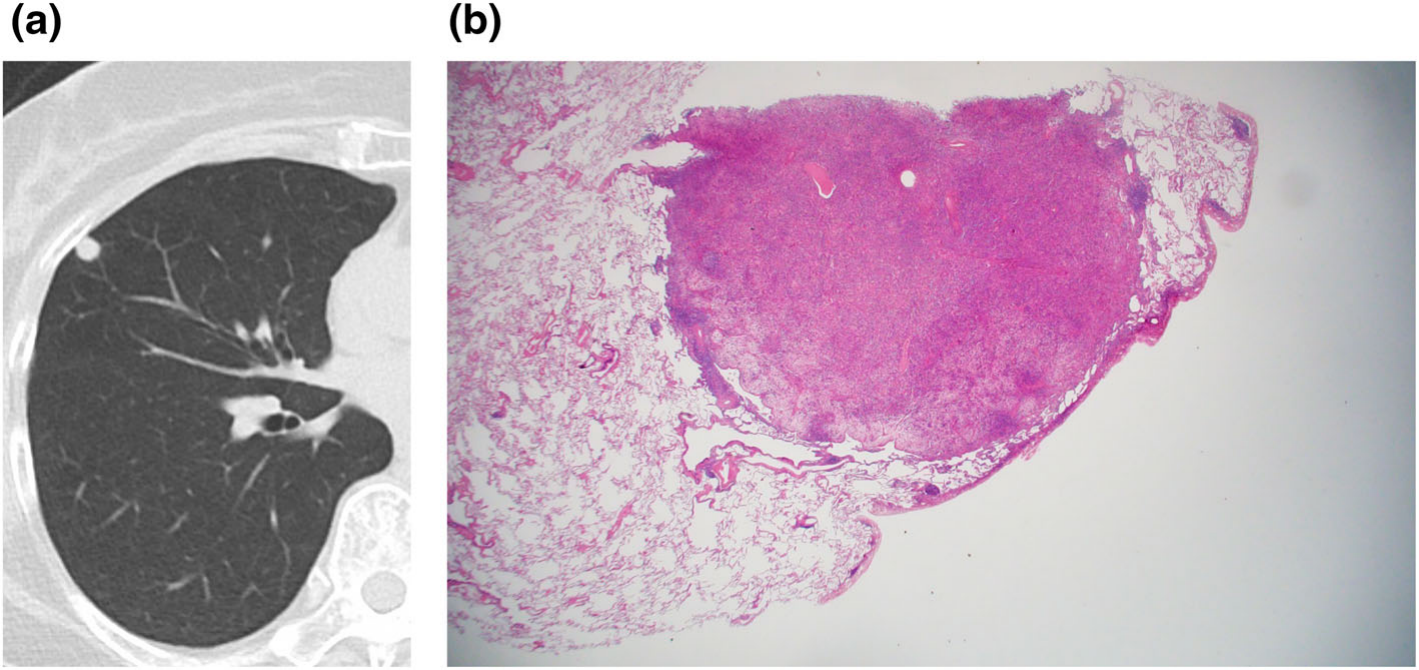
Pulmonary mucosa‐associated lymphoid tissue (MALT) lymphoma in a 58‐year‐old female with underlying breast cancer. (a) Axial chest computed tomography (CT) image shows a 6‐mm sized well‐defined solid subpleural nodule in the right middle lobe. The lesion was misdiagnosed as a metastatic nodule before resection. (b) Microscopic examination of the resected surgical specimen at a low power field shows that the lesion consists mostly of a central solid area with no surrounding lesions consisting of sparse nodules. (c) A more magnified view shows that the lesion consists of a monotonous low‐grade B cells with marked plasmacytic differentiation.

The proportion of central solid area with alveolar destruction and the peripheral area with skip lesions along the bronchovascular bundles varied among lymphoma lesions. Peripherally located neoplastic infiltrates consisted of nodular lesions of varying sizes and shapes and occasional reactive germinal centers, with variable distance among the individual infiltrates. Greater than 10% of the peripheral area was observed in 75% of all cases. The lesions showing the galaxy sign on CT demonstrated a significantly higher proportion of peripheral infiltrates (Figures 2 and 3) compared to those without the galaxy sign (Figure 5) (44.0% vs. 18.8%, *p* = 0.001).

## DISCUSSION

Our study demonstrated that the galaxy sign is also seen on CT of pulmonary MALT lymphoma, other than tuberculosis or sarcoidosis. The galaxy sign was observed in about half of our cohort, with moderate interrater agreement; it was also significantly associated with making a correct impression on CT prior to pathological verification. On pathological analysis, pulmonary MALT lymphoma that showed galaxy sign on CT demonstrated a higher proportion of peripheral lymphoid cell infiltrates.

In our study, the galaxy sign on CT correlated with peripheral aggregates of nodular lymphoid cell infiltrates on pathological analysis. Sarcoidosis and tuberculosis are two diseases that can demonstrate the galaxy sign and perilymphatic nodules on CT.^17^, ^18^ A perilymphatic distribution is defined as “distribution along or adjacent to the lymphatic vessels in the lung”, which more specifically indicates along the bronchovascular bundles, in the interlobular septa, around larger pulmonary veins, and in the pleura.^15^ Pulmonary MALT lymphoma is also known to spread along the bronchovascular bundles, interlobular septa, and along the pleura at the periphery of the lesion.^19^ The bronchus‐associated lymphoid tissue (BALT), from which the pulmonary MALT lymphoma originates, is also known to be found along the bronchovascular bundles.^19^ One study reported that the galaxy sign in pulmonary tuberculosis was only observed in tuberculosis with perilymphatic distribution.^18^ From these observations, it can be inferred that the galaxy sign is not disease‐specific but may be seen in any diseases that show a cluster of perilymphatic nodules. Although the underlying pathology of pulmonary MALT lymphoma is different from sarcoidosis or tuberculosis, the areas involved by the diseases are similar, likely contributing to similar appearances on CT.

Two other studies have also focused on the radiological and pathological correlation of pulmonary MALT lymphoma. In one study, the authors have identified pathological bases for CT findings of air bronchogram, coarse spiculate with different lengths, a well‐marginated halo sign, irregular cavitation, CT angiogram sign, and bronchovascular bundle thickening.^7^ All of these different CT findings were results of different patterns of lymphoid cell infiltration along different structures. In another study, masses/nodules or mass‐like areas of consolidation along bronchovascular regions on CT were clusters of monoclonal lymphocytes with occasional interspersed plasma cells infiltrating along bronchovascular bundles and interlobular regions in the bronchiolar mucosa and alveolar spaces.^20^ Although the galaxy sign was not investigated in that study, our results are consistent with findings in that study, because the galaxy sign on CT is also formed by a certain pattern of lymphoid cell infiltrates in the periphery of the MALT lymphoma lesion.

The fact that the galaxy sign was significantly associated with making a correct first impression on CT implies that although radiologists may not have been able to name or recognize the imaging finding as the galaxy sign, the actual image finding of the galaxy sign may have been unknowingly used by the radiologists in making the correct diagnosis of pulmonary MALT lymphoma. Now that it has been identified and named, radiologists may actively look for this imaging finding to help in the differential diagnosis when reading a chest CT. CT features other than the galaxy sign that were helpful in making a correct impression were the presence of extrapulmonary involvement, multiple lesions, intralesional cavity, bronchocentric distribution, and consolidation type. A combination of these features may be helpful in the diagnosis of pulmonary MALT lymphoma.

The most frequent CT features of pulmonary MALT lymphoma in our study was air bronchogram, which was seen in about 90% of the study cohort. This is in line with other studies, which have reported the frequency of air bronchogram to be 58%–100% in pulmonary MALT lymphoma.^3^, ^4^, ^5^, ^6^, ^7^, ^8^, ^11^ Other frequent CT features included perilesional spicules (79.1%) and bronchocentric distribution (74.4%). One study has reported the presence of “coarse spiculate with different lengths” to be 72.7%,^7^ showing similar results with perilesional spicules in our study. Although a few studies have reported the presence of peribronchovascular thickening in pulmonary MALT lymphoma,^3^, ^7^, ^8^ none of the studies have explicitly used the term “bronchocentric” nor have they focused on the distribution of the lesions. As air bronchogram can be seen in many other pulmonary diseases, including pneumonia and lung adenocarcinoma, recognizing perilesional spicules and bronchocentric distribution of the lesions that show air bronchogram may help narrow the differentials on CT. Another study evaluated the differential diagnosis of pneumonia‐like consolidation pattern of pulmonary MALT lymphoma and lobar pneumonia, both of which show air bronchogram on CT. In that study, bronchiectasis and bulging of the interlobar fissure were two ancillary findings to help differentiate pulmonary MALT lymphoma from lobar pneumonia.^21^

In addition, more than 60% of the cases in our study demonstrated multiplicity and appeared as a nodule/mass pattern rather than consolidation pattern. Multiple nodules and/or masses with air bronchogram that also show perilesional spicules, bronchocentric distribution, or the galaxy sign may be helpful findings in the diagnosis of pulmonary MALT lymphoma on CT.

There are several limitations in our study. First, this was a single‐center, retrospective study with a relatively small number of study subjects. The results would be more convincing if they could be validated by another datasets. However, pulmonary MALT lymphoma is a relatively rare disease, and only 43 subjects could be included over the span of 10 years at a single institution. Future studies with larger number of subjects and multiple centers may help validate the results of our study. Second, only the univariable logistic analyses could be performed for analyses of factors associated with a correct first impression, due to the small number of patients with correct first impression; at least 20 subjects are needed in order to perform multivariable logistic analyses with two variables. Because extrapulmonary involvement is an important factor in making a correct diagnosis for pulmonary MALT lymphoma, multivariate analyses adjusting for the presence of extrapulmonary involvement would have resulted in a more accurate assessment of the usefulness of the galaxy sign in making a correct diagnosis. Third, using the rate of correct first impression for evaluating the usefulness of the galaxy sign is only an indirect way of assessing the usefulness of the galaxy sign. Further studies involving CTs of pulmonary MALT lymphoma as well as other pulmonary pathologies analyzing the sensitivity, specificity, positive predictive value, and negative predictive value of the galaxy sign are needed in order to directly evaluate the usefulness of the galaxy sign in diagnosing pulmonary MALT lymphoma on CT. Fourth, pathological analysis was only done for 28 of 43 patients because only the surgical specimens were reviewed. Because biopsy specimens are usually acquired from the center of the lesion and only represent part of the lesion, the authors felt that biopsy specimens would not be able to sufficiently represent the galaxy sign on pathology. Fifth, almost half of our cohort had incorrect first impression on CT, and the galaxy sign was present in only about 15% of this group. Even with the knowledge of the galaxy sign, most of these patients would still result in incorrect first impression, making pulmonary MALT lymphoma a diagnostic challenge; further studies on useful imaging characteristics for diagnosis of pulmonary MALT lymphoma seems to be necessary.

In conclusion, the galaxy sign can be seen on CT of pulmonary MALT lymphoma with a higher proportion of peripheral lymphoma infiltrates and may be useful in making a correct diagnosis of pulmonary MALT lymphoma.

## AUTHOR CONTRIBUTIONS

Conceptualization, Kyongmin S. Beck; methodology, Yeongran Song, Yeoun Eun Sung, Suyon Chang, Kyongmin S. Beck; formal analysis, Yeongran Song, Yeoun Eun Sung, Kyongmin S. Beck; investigation, all authors; resources, all authors; data curation, Yeongran Song, Yeoun Eun Sung; writing—original draft preparation, all authors; writing—review and editing, Yeongran Song, Yeoun Eun Sung, Kyongmin S. Beck; visualization, Yeongran Song, Yeoun Eun Sung, Kyongmin S. Beck; supervision, Kyongmin S. Beck, Gyeong Sin Park; project administration, Kyongmin S. Beck. All authors have read and agreed to the published version of the manuscript.

## CONFLICT OF INTEREST STATEMENT

The authors have no conflicts of interest to disclose.

## Supporting information


**Data S1.** Supporting Information

## Data Availability

The datasets generated or analyzed during the study are available from the corresponding author on reasonable request.
