# Supplementary material for: Radiological and pathological analysis of the galaxy sign in patients with pulmonary mucosa‐associated lymphoid tissue (MALT) lymphoma
Source: Thorac Cancer. 2023 Jul 6;14(24):2459–66. doi: 10.1111/1759-7714.15029 (PMC10447172; doi:10.1111/1759-7714.15029)
Supplement: Supplementary file 1 — Data S1. Supporting Information [file TCA-14-2459-s001.docx]

**Supplementary material**

**1. Defition of Terms with Example Images**

**“galaxy sign”**


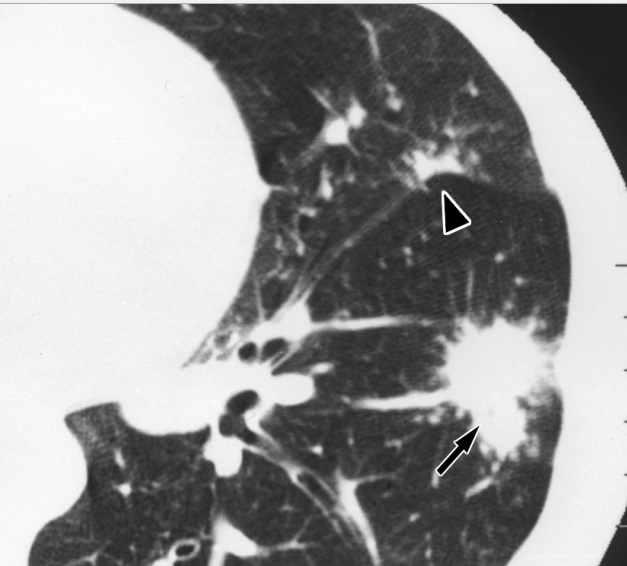


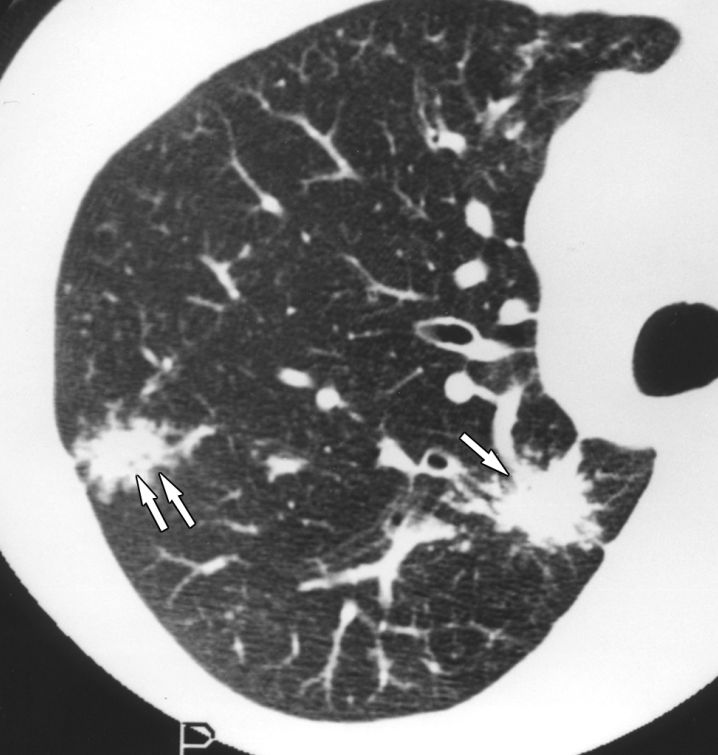


“The large parenchymal nodules consisted of numerous small nodules and showed irregular margins (Figs. 1–3). In the periphery of the large nodules, each constituent small nodule had a relatively distinct margin. Small low attenuations ots were seen in the large nodules (Figs. 1B and 1C). This characteristic appearance of large parenchymal nodules resembled a galaxy that is a vast collection of millions and occasionally billions of stars (Fig. 4). This sign was recognized in all 16 patients. Smooth boundaries in three of the large nodules caused investigators to disagree about their classification as the sarcoid galaxy sign. However, galaxy appearances were also evident in portions of these questionably large nodules (Fig. 2B). After reaching a consensus, we categorized these large nodules with partially smooth boundaries as sarcoid galaxies.” (1)


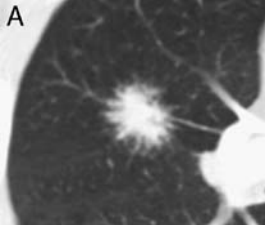


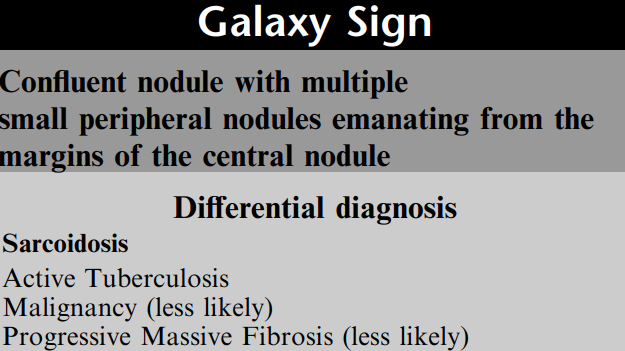
 (2)


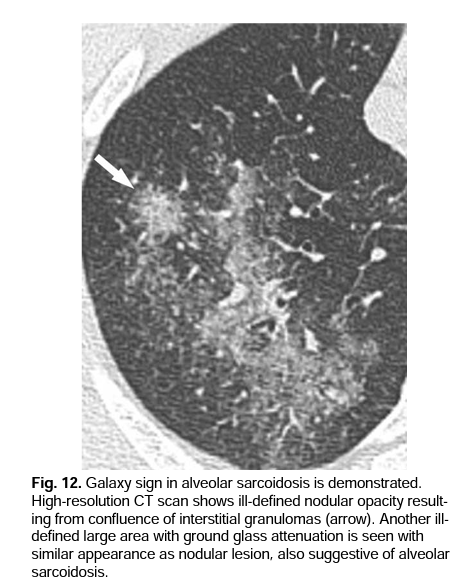
(3)


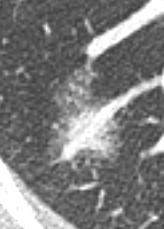

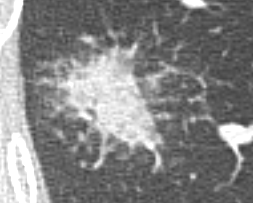


**Air-bronchogram**

“An air bronchogram is a pattern of air-filled (low-attenuation) bronchi on a background of opaque (high-attenuation) airless lung”(4)


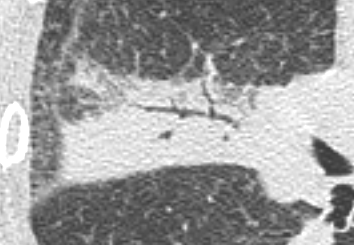


**Perilesional spicules**

Nodular or smooth interstitial thickening around the nodule/mass or consolidation, seen as irregular spicules


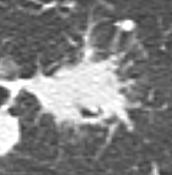

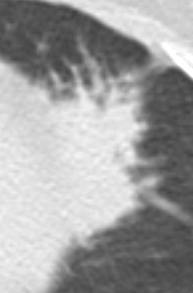


**Separate interlobular septal thickening**

Interlobular septal thickening separate from nodule/mass/consolidation


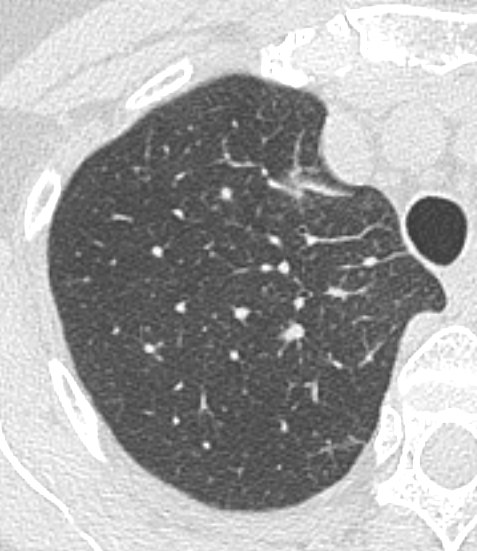


**Cavity**

“A cavity is a gas-filled space, seen as a lucency or low-attenuation area, within pulmonary consolidation, a mass, or a nodule”(4)


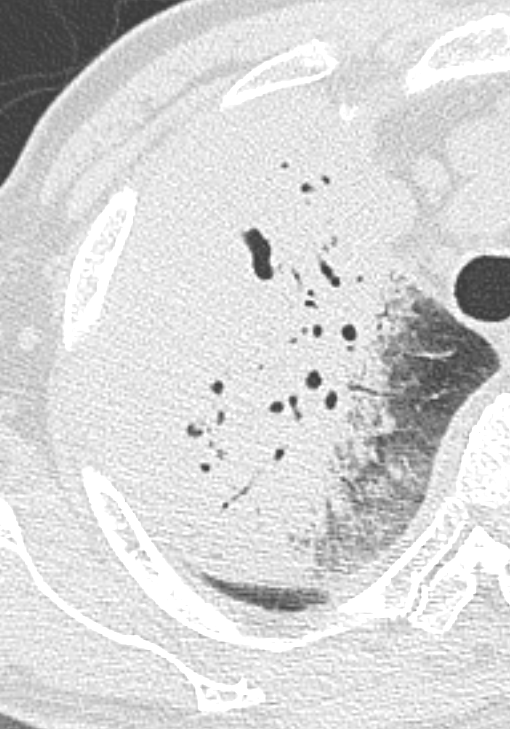

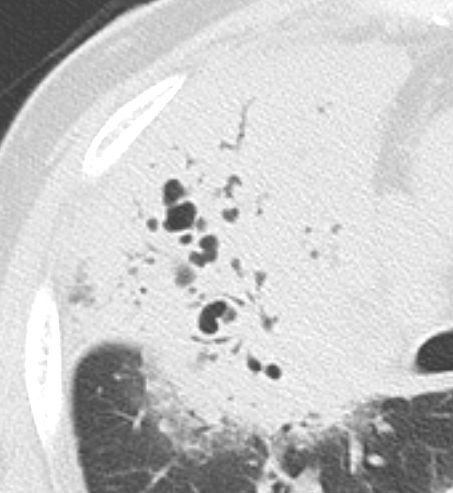


**Bronchocentric distribution**


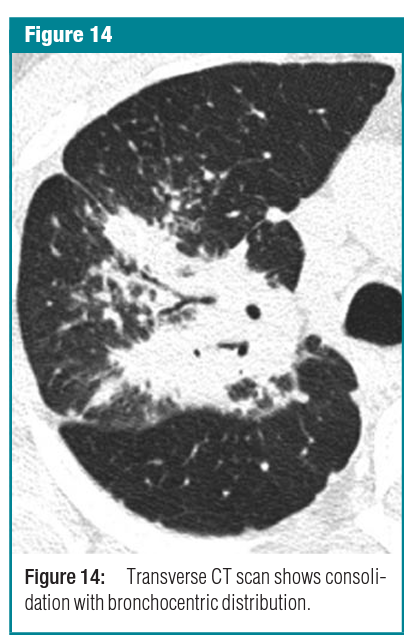


This descriptor is applied to disease that is conspicuously centered on macroscopic bronchovascular bundles.(4)

**References:**

1. Nakatsu M, Hatabu H, Morikawa K, Uematsu H, Ohno Y, Nishimura K, et al. Large Coalescent Parenchymal Nodules in Pulmonary Sarcoidosis: “Sarcoid Galaxy” Sign. American Journal of Roentgenology. 2002;178(6):1389-93.

2. Aikins A, Kanne JP, Chung JH. Galaxy Sign. Journal of Thoracic Imaging. 2012;27(6):W164.

3. Park HJ, Jung JI, Chung MH, Song SW, Kim HL, Baik JH, et al. Typical and atypical manifestations of intrathoracic sarcoidosis. Korean J Radiol. 2009;10(6):623-31.

4. Hansell DM, Bankier AA, MacMahon H, McLoud TC, Müller NL, Remy J. Fleischner Society: Glossary of Terms for Thoracic Imaging. Radiology. 2008;246(3):697-722.

**2. Detailed methods of molecular testing**

**IgH Gene Rearrangement Analyzed With PCR**

Genomic DNA was extracted from the formalin-fixed paraffin-embedded (FFPE) tissue block sections with Maxwell® 16 FFPE plus LEV DNA Purification kit (Promega), according to the manufacturer’s protocol. PCR was used to amplify a fragment of the human immunoglobulin heavy chain (IgH) gene. The BIOMED-2 standardized primer system was used to detect the IgH gene rearrangement. The PCR cycling conditions were: predenaturation at 95˚C for 7 min, 40 cycles of denaturation at 95˚C for 45 sec, annealing at 60˚C for 45 sec, and extension at 72˚C for 90 sec, with a final extension at 72˚C for 10 min. The PCR-amplified fragments were loaded onto an ABI 3500xl (Foster City, CA) Genetic Analyzer for capillary electrophoresis. The GeneMapper ID V4.1 software was used for the data analysis. If there were one or two relatively high peaks in the polyclonal background within the expected size range, and the height of the highest peak was three times that of the third highest peak, the result was defined as positive.

**Detection of MALT1 Gene Rearrangements**

Fluorescence in situ hybridization (FISH) of MALT1 gene was performed, using Vysis LSI MALT1 Dual Color Break Apart Rearrangement Probe (Vysis), according to the manufacturer’s instructions. The hybridized signals for each probe were evaluated in the interphase nuclei of 100 cells. The cutoff values for positive were set at 10 %.

**mRNA in situ hybridization**

Kappa and Lambda light chain mRNA detection was performed using INFORM **®** cytoplasmic Kappa / Lambda mRNA Probe (Roche Ventana Medical Systems), according to the manufacturer’s instructions. In brief, the sections are baked (32 min at 60°C) and then deparaffinized, followed by target retrieval (24 min at 97°C for tissues) and protease treatment (16 min at 37°C). The probes were then hybridized for 2 hours at 43°C followed by RNAscope amplification. Positive signals were detected as blue cytoplasmic deposits.
